# IFNγ and TNFα synergistically induce apoptosis of mesenchymal stem/stromal cells via the induction of nitric oxide

**DOI:** 10.1186/s13287-018-1102-z

**Published:** 2019-01-11

**Authors:** Xiaolei Li, Bingxue Shang, Ya-nan Li, Yufang Shi, Changshun Shao

**Affiliations:** The First Affiliated Hospital of Soochow University and State Key Laboratory of Radiation Medicine and Protection, Institutes for Translational Medicine, Soochow University, Suzhou, 215123 Jiangsu China

**Keywords:** Mesenchymal stem/stromal cells, Inflammation, iNOS, Nitric oxide, Autophagy, Apoptosis

## Abstract

**Background:**

Mesenchymal stem/stromal cells (MSCs) have been widely used to treat various inflammatory diseases. The immunomodulatory capabilities of MSCs are usually licensed by inflammatory cytokines and may vary depending on the levels and the types of inflammatory cytokines. However, how the inflammatory microenvironment affects the fate of MSCs remains elusive. Here we characterized the molecular mechanism underlying the apoptosis of mouse MSCs triggered by the synergistic action of IFNγ and TNFα.

**Methods:**

We isolated and expanded MSCs by flushing the femoral and tibial bone marrow of wild-type, iNOS^−/−^, and Fas^−/−^ mice. BM-MSCs were treated with IFNγ and TNFα in vitro, and cell viability was evaluated by a CCK-8 kit. Apoptosis was assessed by Annexin V/propidium iodide-stained flow cytometry. Expression of genes related to apoptosis and endoplasmic reticulum (ER) stress was measured by reverse transcription-polymerase chain reaction (RT-PCR). Apoptosis and autophagy-related proteins were examined by Western blot analysis.

**Results:**

IFNγ and TNFα synergistically trigger apoptosis of mouse BM-MSCs. The two cytokines were shown to stimulate the expression of inducible nitric oxide synthase (iNOS) and consequently the generation of nitric oxide (NO), which is required for the apoptosis of mouse BM-MSCs. The two cytokines similarly induced apoptosis in Fas^−/−^ BM-MSCs. iNOS and NO were shown to upregulate Fas in mouse MSCs and sensitize them to Fas agonist-induced apoptosis. Moreover, NO stimulated by IFNγ/TNFα impairs autophagy, which aggravates ER stress and promotes apoptosis.

**Conclusions:**

IFNγ/TNFα-induced apoptosis in mouse MSCs is mediated by NO. Our findings shed new light on cytokine-induced apoptosis of MSCs and have implications in MSC-based therapy of inflammatory diseases.

**Electronic supplementary material:**

The online version of this article (10.1186/s13287-018-1102-z) contains supplementary material, which is available to authorized users.

## Background

Mesenchymal stem/stromal cells (MSCs) are multipotent stem cells that exist in almost all types of tissues, including bone marrow, umbilical cord, muscle, fat, and dermis [1]. Depending on the stimuli and the culture conditions employed, MSCs are capable of differentiating into osteoblasts, adipocytes, and chondrocytes [2]. In addition to their potential to differentiate into multiple cell lineages, MSCs also acquire potent immunoregulatory function when properly stimulated, which makes them suitable and highly effective for clinical applications in treating a variety of human disease, such as graft-versus-host disease (GvHD) and systemic lupus erythematosus (SLE) [1–3].

The inflammatory microenvironment plays a crucial role in the acquisition of the immunoregulatory capability by MSCs [4]. Mechanistic studies exploring the immunosuppressive effects of MSCs on T cells have revealed that a combination of interferon (IFN)-γ and any of three other pro-inflammatory cytokines, tumor necrosis factor (TNF)-α, interleukin (IL)-1α, and IL-1β, can activate MSCs to exert immunosuppressive effects in mice [5]. These cytokine combinations provoke the expression of high levels of several chemokines and inducible nitric oxide synthase (iNOS) by MSCs [5]. The T cells are recruited to the proximity of the MSCs by chemokines and are killed by nitric oxide (NO). Unlike murine MSCs which utilize iNOS, human MSCs employ indoleamine 2,3-dioxygenase (IDO), the tryptophan-catabolic enzyme, to suppress T cells [1].

While exogenous MSCs have been widely used to repair tissue damage and to control inflammation via the production of chemokines and NO or IDO, the infused MSCs tend to have a very limited lifespan [6]. How those MSCs disappear in vivo remains largely elusive. One study showed that when exposed to IFNγ/TNFα mouse MSCs could undergo apoptosis mediated by Fas (CD95/Apo-1) and were thus compromised in their bone-repairing function [7]. IFNγ plus TNFα were also shown to induce autophagy in MSCs and inhibition of autophagy could augment the immunosuppressive effect on T cells by MSCs [8, 9]. However, survival of MSCs was recently shown to be dispensable for the immunosuppressive effect [10]. Instead, MSCs are actively induced to undergo perforin-dependent apoptosis by recipient cytotoxic cells and the apoptosis of MSCs is essential for their immunosuppression in a murine model of GvHD. These studies suggest that fate of MSCs may modulate the therapeutic effects of MSCs in a context-dependent manner. Since iNOS is drastically induced by IFNγ plus TNFα in MSCs and NO is responsible for the cell cycle arrest and apoptosis of T cells, we hypothesized that NO might also play a role in the death of murine MSCs when exposed to IFNγ and TNFα. The finding that NO can impair pro-survival autophagy also renders support to this notion [11]. We therefore investigated apoptosis induced by IFNγ plus TNFα in cultured mouse bone-marrow (BM)-MSCs (BM-MSCs). We found that NO indeed mediates the death of murine MSCs upon exposure to IFNγ and TNFα.

## Methods

### MSC isolation and culture

MSCs were generated using our previously described protocol [5]. Briefly, tibia and femur bone marrow of 6-week-old wild-type, iNOS^−/−^, and Fas^−/−^ mice was harvested. Cells were cultured in DMEM supplemented with 10% FBS, 2 mM glutamine, 100 U/ml penicillin, and 100 μg/ml streptomycin (complete medium, all from Invitrogen, Carlsbad, CA, USA). All non-adherent cells were removed after 24 h, and adherent cells were maintained. Medium was changed every 3 days. To obtain MSC clones, cells at confluence were harvested and seeded into 96-well plates by limited dilution. Individual clones were then picked and expanded. These MSCs were capable of differentiating into adipocytes and osteocytes under the respective differentiation conditions. Cells were used before the 20th passage.

### Cell viability assay

Cell viability was determined using a CCK-8 Kit (Dojindo, Japan). Cells were seeded into 96-well plates at 2 × 10^3^ cells/well and cultured for 24 h at 37 °C with 5% CO_2_. After treatment with PBS as a control or IFNγ and TNFα at varying concentrations for the indicated times, 10 μl of CCK-8 solutions was added to each well of the plate. The cells were incubated for one more hour, and the absorbance of the samples (450 nm) was determined by using a scanning multi-well spectrophotometer. The cell viability was calculated using the following formula: relative cell viability (%) = (absorbance_450 nm_ of treated group − absorbance_450 nm_ of blank)/(absorbance_450 nm_ of control group − absorbance_450 nm_ of blank). The results from three independent experiments in triplicates are presented.

### Real-time PCR

Total RNA from each sample was extracted by the Trizol reagent (Thermo Fisher). Purity was addressed by the absorbance ratio at 260 and 280 nm. First-strand cDNA synthesis was performed using PrimeScript™ RT Master Mix according to the manufacturer’s instructions (TaKaRa Biotech, Dalian, China). The levels of mRNA of genes of interest were measured by real-time PCR (7900 HT by Applied Biosystems, Foster City, CA, USA) using SYBR Green Master Mix (TaKaRa Biotech, Dalian, China). Total amount of mRNA was normalized to endogenous β-actin mRNA. The primers of the target genes were designed as shown in Table 1.

**Table 1:** Gene-specific primers for qRT-PCR

Genes	Oligonucleotide sequence (5′–3′)
β-actin	F: CAACGAGCGGTTCCGATG
	R: GCCACAGGATTCCATACCCA
Atf4	F: CGGGTGTCCCTTTCCTCTTC
	R: TGAAGAGCGCCATGGCTTAG
Bip/GRP78	F: GTGTGTGAGACCAGAACCGT
	R: ACAGTGAACTTCATCATGCCG
Erdj4/Dnajb9	F: GGGGCGCACAGGTTATTAGAA
	R: TCTGAGGCAGACTTTGGCAC
Chop	F: GGAACCTGAGGAGAGAGTGTT
	R: AAGGTGAAAGGCAGGGACTC
Xbp1	F: GCAGCAAGTGGTGGATTTGG
	R: CCTTACTCCACTCCCCTTGG
Fas	F: GCTTGCTGGCTCACAGTTAAG
	R: AGGTTGGTGTACCCCCATTC
Bcl2	F: CACCCCTGGTGGACAACATC
	R: ATAGTTCCACAAAGGCATCCCAG
Bcl2l11	F: GCCAGGCCTTCAACCACTAT
	R: TGCAAACACCCTCCTTGTGT
Bax	F: TGCTAGCAAACTGGTGCTCA
	R: AGTAGGAGAGGAGGCCCAGC

*F* forward primer, *R* reverse primer

### Western blotting

Cells were washed twice with ice-cold PBS, harvested and lysed in the RIPA buffer (Millipore, Temecular, CA, USA) containing a cocktail of protease inhibitors (Roche, Nutley, NJ, USA) and PMSF for 30 min on ice. Lysates were clarified by centrifugation at 16000×*g* for 15 min and heated in sodium dodecyl sulfate sample buffer at 95 °C for 10 min. Protein concentration of the supernatant was determined by the Bradford assay (Bio-Rad, Hercules, CA, USA). Protein samples were separated on a polyacrylamide gel, and separated proteins were electroblotted onto polyvinylidene difluoride membranes. Specific proteins were revealed by mouse and rabbit antibodies by overnight incubation at 4 °C, followed by chemiluminescent detection according to the manufacturer’s instructions.

### Annexin-V/propidium iodide flow cytometric analysis

The apoptosis induced by cytokines and/or *S*-nitroso-*N*-acetyl-penicillamine (SNAP) was assessed by staining cells with an Annexin V-fluorescein isothiocyanate (APC) Apoptosis Detection Kit (BD Biosciences). The cells were collected and then washed with cold PBS twice. Then, they were resuspended in 100 μl of Annexin V binding buffer and incubated with 5 μl of APC-conjugated Annexin V and 5 μl of propidium iodide for 15 min in the dark. Annexin V binding buffer (200 μl) was then added to each tube. Finally, the cells were examined using a BD FACS-Canto II flow cytometer (BD Biosciences, CA). All experiments were performed in triplicate and repeated three times independently.

### Statistical analysis

The data are presented as the means ± SD of three independent experiments. For the determination of statistical significance, a one-way analysis of variance (ANOVA) and a two-way ANOVA were performed using Prism software (version 7.0, GraphPad Software, La Jolla, CA). *P* values < 0.05 were considered statistically significant.

## Results

### IFNγ and TNFα synergistically trigger apoptosis in mouse BM-MSCs

**Fig. 1:**
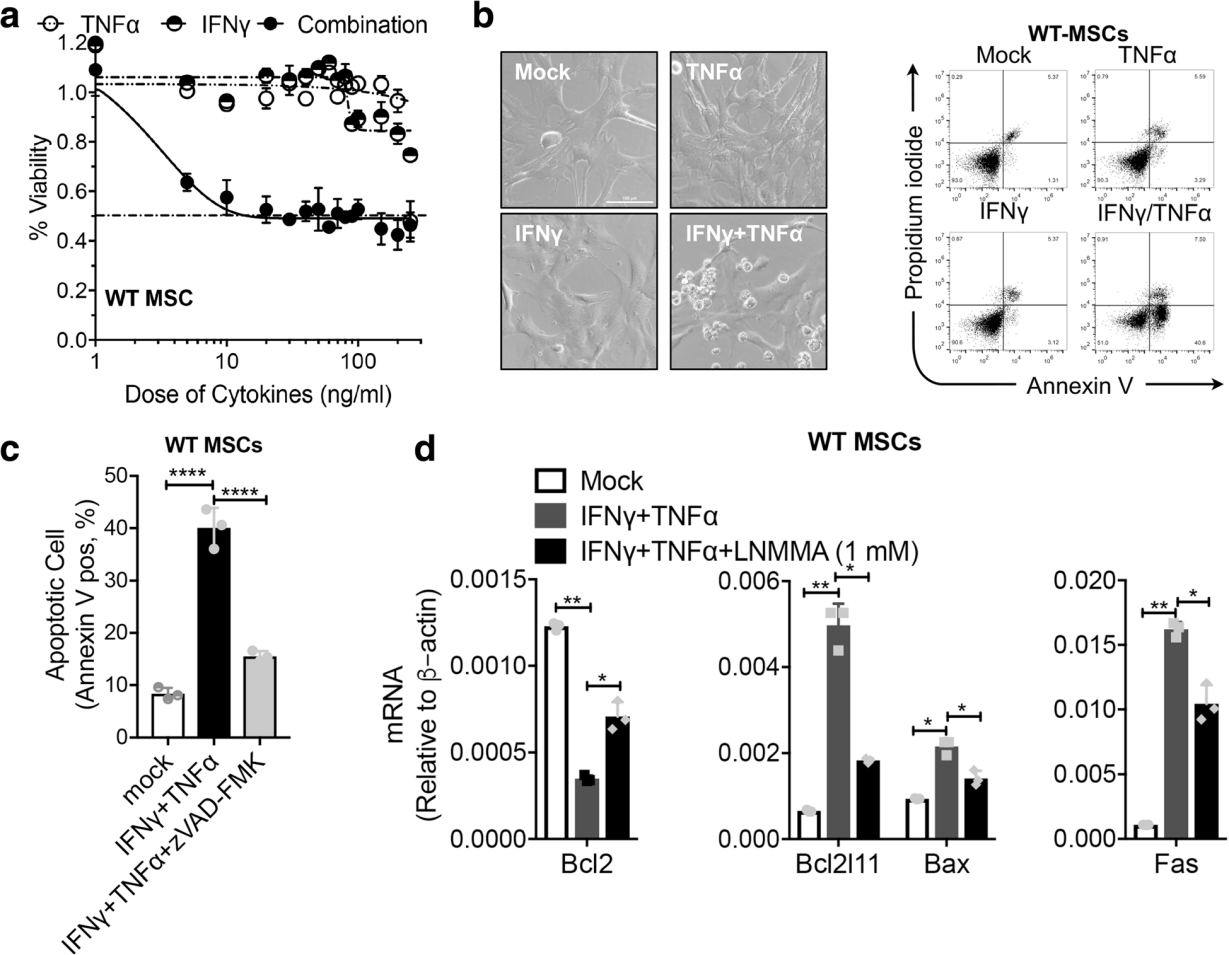
IFNγ and TNFα synergistically induced apoptosis in mouse BM-MSCs. **a** Percentage of wild-type BM-MSC cell survival after treatment with various concentrations of TNFα or IFNγ alone or a combination of these two cytokines. **b** Wild-type BM-MSCs were treated with IFNγ/TNFα (10 ng/ml each) for 48 h. Apoptotic cells were analyzed using flow cytometry. The percentage of Annexin V-positive cells relative to untreated controls is indicated in a bar chart. **c** Wild-type BM-MSCs were treated with IFNγ/TNFα (10 ng/ml each) in the absence or presence (1 h preincubation) of zVAD-FMK for 48 h. Apoptotic cells were analyzed using flow cytometry. The percentage of Annexin V-positive cells relative to untreated controls is indicated in a bar chart. **d** BM-MSCs were treated with IFNγ/TNFα (10 ng/ml each) in the absence or presence (1 h pre-incubation) of L-NMMA (1 mM) for 24 h. Pro-apoptotic and anti-apoptotic transcripts were quantified by real-time PCR. *P < 0.05, **P < 0.01, ****P < 0.0001

To investigate whether inflammatory cytokines induce apoptosis of MSCs, we cultured BM-MSCs in vitro with TNFα, IFNγ, or the two in combination. Treatment of mouse BM-MSCs with TNFα or with IFNγ alone did not substantially affect their viability (Fig. 1a). However, the two in combination significantly reduced MSC viability and induced massive cell death (Fig. 1a, b), which could be inhibited by the caspase inhibitor Z-VAD-FMK (Fig. 1c). Among the important regulators of apoptosis are the pro- and anti-apoptotic proteins of the Bcl-2 family and the pro-apoptotic multidomain proteins Bax and Bak are needed for apoptosis mediated by mitochondria [12]. Oligomerization of Bax and Bak leads to formation of membrane pores through which apoptotic mediators, such as cytochrome *c,* are released to the cytoplasm and/or apoptosis-inducing factor (AIF) to the nucleus [12]. Once in the cytoplasm, cytochrome *c* interacts with apoptotic protease activating factor to form the apoptosome, leading to pro-caspase cleavage and activation and subsequent cell death. We examined the effect of IFNγ/TNFα treatment on the expression of representative pro- and anti-apoptotic genes in MSCs by quantitative RT-PCR analysis. As shown in Fig. 1d, treatment of mouse BM-MSCs with IFNγ/TNFα led to higher expression of pro-apoptotic genes and lower expression of anti-apoptotic genes in BM-MSCs compared with controls. These data indicate that IFNγ/TNFα synergistically induce BM-MSC apoptosis.

### BM-MSC apoptosis induced by IFNγ and TNFα is mediated by NO

**Fig. 2:**
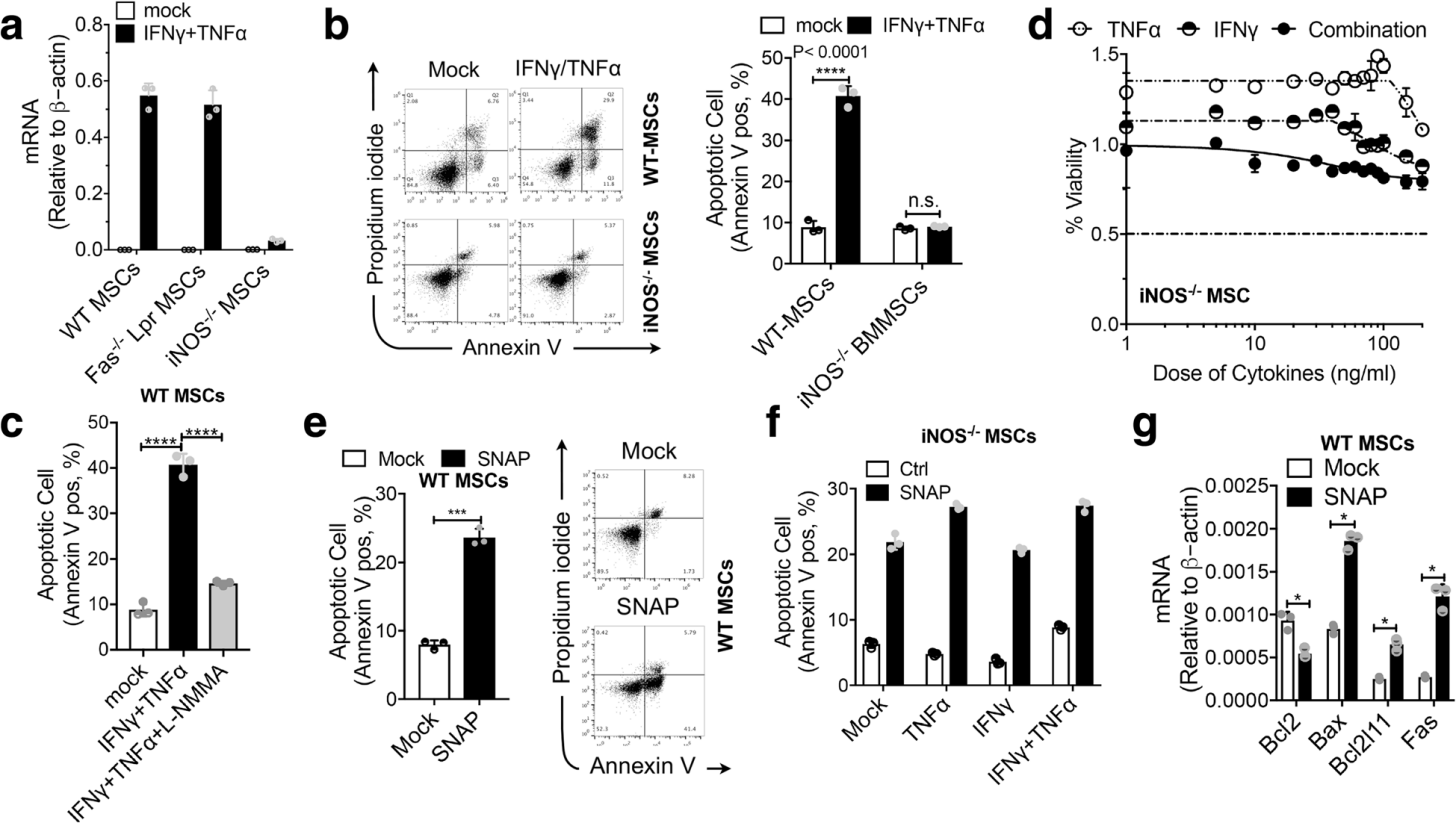
BM-MSC apoptosis induced by IFNγ and TNFα is mediated by NO. **a** Wild-type, Fas^−/−^, and iNOS^−/−^ BM-MSCs were treated with IFNγ plus TNFα for 24 h or for the indicated time. The levels of iNOS expression were quantified by real-time PCR. **b** Wild-type and iNOS^−/−^ BM-MSCs were treated with IFNγ/TNFα (10 ng/ml each) for 48 h. Apoptotic cells were analyzed using flow cytometry. The percentage of Annexin V-positive cells relative to untreated controls is indicated in a bar chart. **c** Wild-type BM-MSCs were treated with IFNγ/TNFα (10 ng/ml each) in the absence or presence (1 h pre-incubation) of L-NMMA (1 mM) for 48 h. Apoptotic cells were analyzed using flow cytometry. The percentage of Annexin V-positive cells relative to untreated controls is indicated in a bar chart. **d** Percentage of iNOS^−/−^ BM-MSC survival after treatment with various concentrations of different cytokines. **e** Wild-type MSCs were treated with SNAP (0.5 mM) for 48 h. Apoptotic cells were analyzed using flow cytometry. The percentage of Annexin V-positive cells relative to untreated controls is indicated in a bar chart. **f** iNOS^−/−^ BM-MSCs were treated with the indicated conditions for 48 h. Apoptotic cells were analyzed using flow cytometry. The percentage of Annexin V-positive cells relative to untreated controls is indicated in a bar chart. **g** Wild-type BM-MSCs were treated with SNAP; pro-apoptotic and anti-apoptotic transcripts were quantified by real-time PCR. *P < 0.05, **P < 0.01, ***P < 0.001, ****P < 0.0001

We examined the effects of TNFα, IFNγ, or the two in combination on the expression of NOS in MSCs and revealed that the level of iNOS was synergistically enhanced by IFNγ/TNFα, which corresponded to their ability to induce cell death (Fig. 2a, b). Endogenous NO is synthesized from L-arginine by a family of NOS in a two-step oxidation process. An excess of NO resulting in nitrosative stress is believed to play a causal role in demise of several type of cells, including neuronal cells [13], T cells [14], dendritic cells [15], and microglial cells [16]. We therefore tested whether NO is a crucial mediator of IFNγ/TNFα-induced BM-MSC apoptosis. Notably, blocking iNOS activity by the NOS inhibitor N^G^-monomethyl-L-arginine (L-NMMA) could protect BM-MSCs from IFNγ/TNFα-induced cell death (Fig. 2c). The increased apoptosis induced by IFNγ/TNFα was also prevented in BM-MSCs derived from iNOS^−/−^ mice (Fig. 2b–d). Collectively, these results demonstrated that NO plays a critical role in IFNγ/TNFα-induced apoptosis in mouse BM-MSCs.

To further substantiate this notion, we examined whether direct application of NO also induces apoptosis in MSCs via mitochondrial pathway. BM-MSCs were treated with the NO donor *S*-nitroso-*N*-acetyl-penicillamine (SNAP). As shown in Fig. 2e, this treatment caused cell death in BM-MSCs not treated with IFNγ/TNFα. SNAP had similar effect on iNOS^−/−^ BM-MSCs independent of IFNγ/TNFα (Fig. 2f). Death was also accompanied by Bax and Bak activation and activation of caspase-3 (data not shown). Notably, assessment of apoptotic-related gene expression in BM-MSCs revealed that SNAP led to higher expression of pro-apoptotic genes and lower expression of anti-apoptotic genes in BM-MSCs compared with controls (Fig. 2g). These results indicate that NO mediates the cytotoxic effect of IFNγ/TNFα on BM-MSCs.

### Fas is dispensable for the IFNγ/TNFα-induced BM-MSC apoptosis

**Fig. 3:**
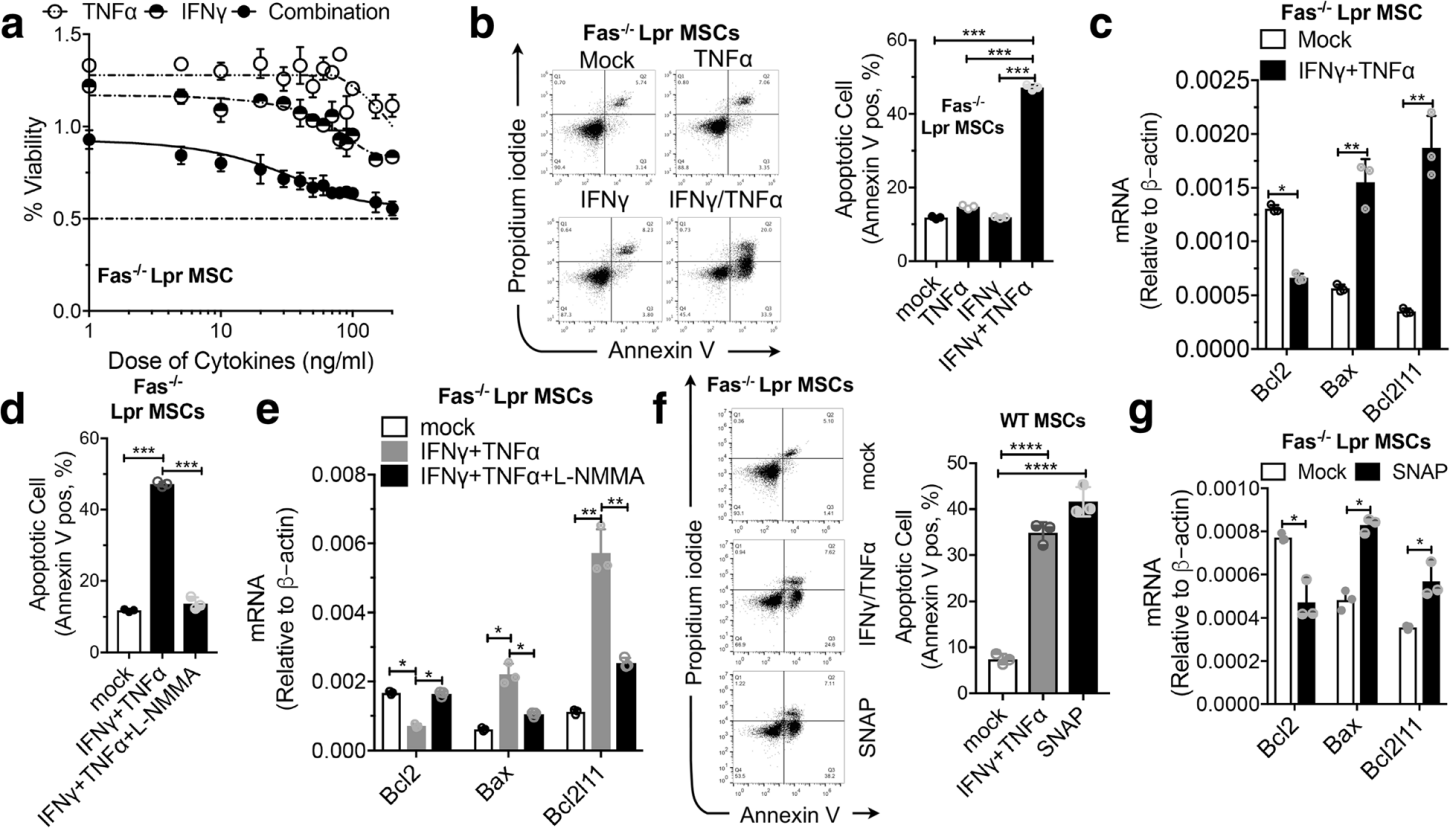
Fas is not required for IFNγ/TNFα-induced apoptosis in MSCs. **a** Percentage of Fas^−/−^ BM-MSC cell survival after treatment with various concentrations of different cytokines. **b** Fas^−/−^ BM-MSCs were treated with the indicated cytokines for 48 h, apoptotic cells were analyzed using flow cytometry. The percentage of Annexin V-positive cells relative to untreated controls is indicated in a bar chart. **c** Fas^−/−^ BM-MSCs were treated with the indicated cytokines for 24 h; pro-apoptotic and anti-apoptotic transcripts were quantified by real-time PCR. **d** Fas^−/−^ BM-MSCs were treated with IFNγ/TNFα (10 ng/ml each) in the absence or presence (1 h pre-incubation) of L-NMMA (1 mM) for 48 h. Apoptotic cells were analyzed using flow cytometry. The percentage of Annexin V-positive cells relative to untreated controls is indicated in a bar chart. **e** Fas^−/−^ BM-MSCs were treated as described in **d**. Pro-apoptotic and anti-apoptotic transcripts were quantified by real-time PCR. **f** Fas^−/−^ BM-MSCs were treated with SNAP (0.5 mM) or IFNγ/TNFα (10 ng/ml each) for 48 h. Apoptotic cells were analyzed using flow cytometry. **g** Fas^−/−^ BM-MSCs were treated with SNAP (0.5 mM) for 24 h; pro-apoptotic and anti-apoptotic transcripts were quantified by real-time PCR. *P < 0.05, **P < 0.01, ***P < 0.001

Fas is a classical type I transmembrane receptor and belongs to the TNF receptor superfamily [17]. It was reported that Fas internalization and clustering mediate the apoptosis in BM-MSC by activation of caspase-8 or caspase-3 in the presence of IFNγ/TNFα [7]. We further validated whether Fas signaling is required for IFNγ/TNFα-induced BM-MSC apoptosis using Fas^−/−^ BM-MSCs. Unexpectedly, these two pro-inflammatory cytokines in combination could markedly reduce cell viability (Fig. 3a) and induce massive cell death in Fas^−/−^ BM-MSCs (Fig. 3b). These data suggest that Fas receptor may not be required for IFNγ/TNFα-induced BM-MSC apoptosis. Quantitative RT-PCR analysis also revealed increased expression of pro-apoptotic genes and reduced expression of anti-apoptotic genes in Fas^−/−^ BM-MSCs (Fig. 3c).

To further investigate whether apoptosis in Fas^−/−^ BM-MSCs triggered by the combination of IFNγ/TNFα depends on NO production, we examined the effect of IFNγ/TNFα on the expression of NOS in Fas^−/−^ BM-MSCs and observed that the level of iNOS was also stimulated. Notably, apoptosis in Fas^−/−^ BM-MSCs triggered by the combination of IFNγ/TNFα could also be reversed by pretreatment with the NOS inhibitor L-NMMA (Fig. 3d). Additionally, assessment of the effect of L-NMMA on the expression of apoptosis-related genes in Fas^−/−^ BM-MSCs also revealed that NOS inhibitor not only prevented the IFNγ/TNFα-induced increase in expression of pro-apoptotic genes, but also enhanced the level of anti-apoptotic genes in Fas^−/−^ BM-MSCs (Fig. 3e). Moreover, treatment of Fas^−/−^ BM-MSCs with the NO donor SNAP caused massive cell death (Fig. 3f). Assessment of the effects of SNAP on apoptotic-related gene expression in Fas^−/−^ BM-MSCs revealed that SNAP led to higher expression of pro-apoptotic genes and lower expression of anti-apoptotic genes in BM-MSCs compared with controls (Fig. 3g). These data further suggest that Fas signaling might be not required for the IFNγ/TNFα-induced BM-MSC apoptosis and NO, instead, plays a critical role in apoptosis of BM-MSCs triggered by IFNγ/TNFα.

### NO enhances Fas-mediated apoptosis in BM-MSCs

**Fig. 4:**
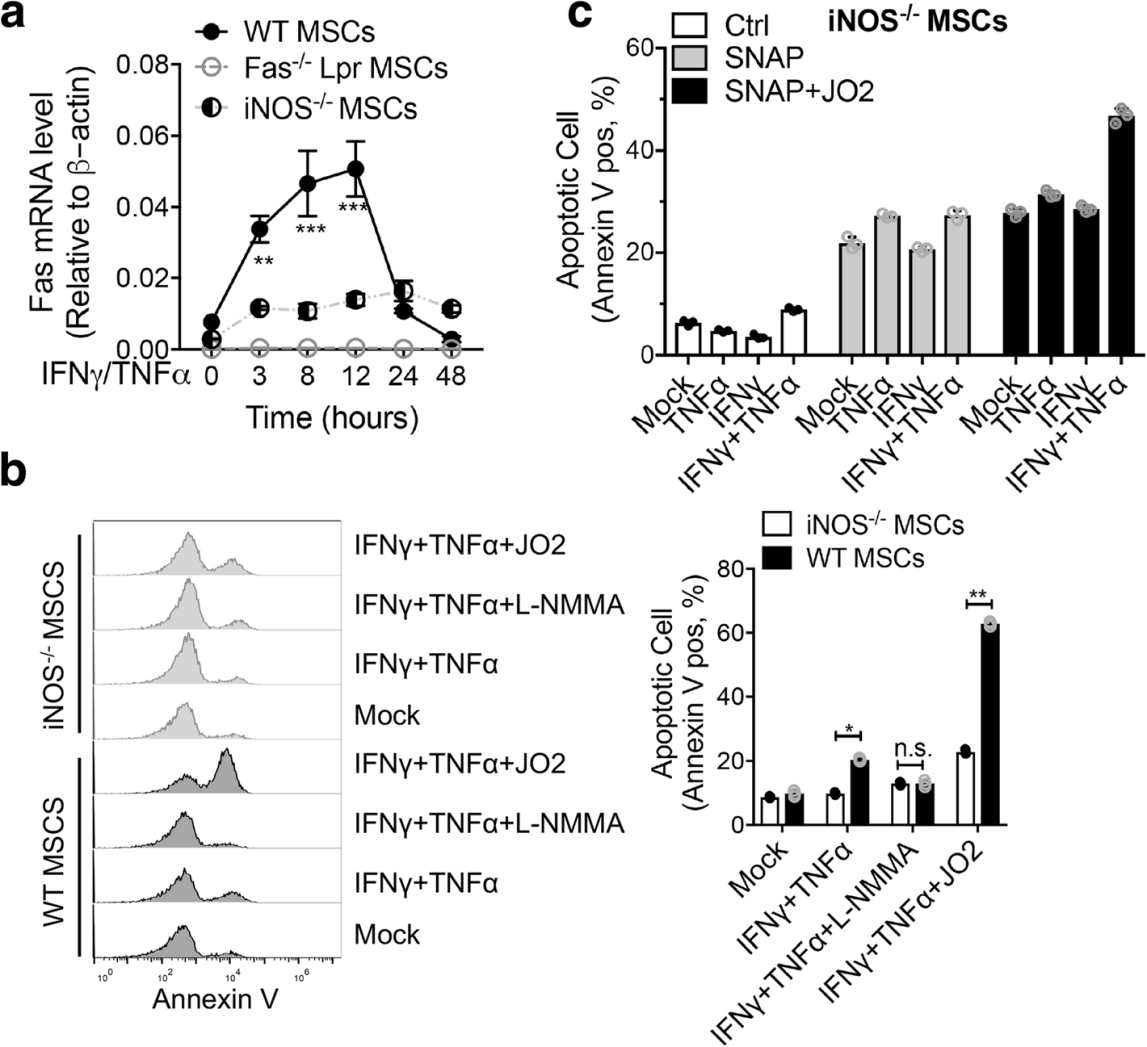
IFNγ/TNFα sensitizes BM-MSCs to Fas-mediated apoptosis iNOS-dependently. **a** Wild-type, Fas^−/−^, and iNOS^−/−^ BM-MSCs were treated with IFNγ/TNFα for 24 h or for the indicated time. The levels of Fas expression were quantified by real-time PCR. **b**, **c** Wild-type, and iNOS^−/−^, BM-MSCs were treated with the indicated conditions for 48 h. Apoptotic cells were analyzed using flow cytometry. The percentage of Annexin V-positive cells relative to untreated controls is indicated in a bar chart. *P < 0.05, **P < 0.01, ***P < 0.001

Fas has been recognized as a death receptor, particularly in mediating non-specific T cell cytotoxicity and activation-induced cell death (AICD) in the peripheral immune system [18]. However, the induction of apoptosis by IFNγ/TNFα in Fas^−/−^, but not in iNOS^−/−^, BM-MSCs argues against Fas playing a critical role in the apoptosis of BM-MSCs. Nevertheless, NO is known to serve as an intracellular second messenger to modify gene expression, and protein modification by *S*-nitrosylation is a common mechanism of NO-mediated signal transduction [19]. The role of NO on Fas receptor expression has been reported. The mechanism involved is NO inhibits Yin Yang 1 (YY1) DNA-binding activity through *S*-nitrosation and consequently results in upregulation of Fas expression [20, 21]. Thus, we next examined whether NO contributes to the IFNγ/TNFα-induced sensitization of mouse BM-MSCs to Fas-mediated apoptosis. As shown in Figs. 1d and 2g, IFNγ/TNFα-mediated upregulation of Fas expression in BM-MSCs was blockaded by NOS inhibitor, but restored by the NO donor (SNAP, 200 μM). Notably, the level of Fas induced by IFNγ/TNFα was significantly reduced in iNOS^−/−^ MSCs, compared with wild-type MSCs (Fig. 4a). These results indicate Fas induction by IFNγ/TNFα in MSCs is possibly dependent on NO. We then examined the sensitivity of wild-type and iNOS^−/−^ BM-MSCs to Fas-mediated apoptosis using Fas agonistic antibody Jo2. As shown in Fig. 4b, apoptosis induced by a combination of IFNγ/TNFα was further enhanced by Jo2. As expected, sensitization to Fas-mediated apoptosis by IFNγ/TNFα was significantly decreased in iNOS^−/−^ MSCs. The role of the NO in the IFNγ/TNFα-mediated sensitization to Fas-induced apoptosis was corroborated by the use of SNAP, an exogenous source of NO that mimics the production of NO by iNOS (Fig. 4c). Our results herein with BM-MSCs clearly demonstrated that NO induced by IFNγ/TNFα, instead of protecting MSCs from Fas-induced apoptosis, synergized with Jo2 to induce apoptosis.

### Blockade of autophagy by iNOS sensitizes BM-MSCs to apoptosis

**Fig. 5:**
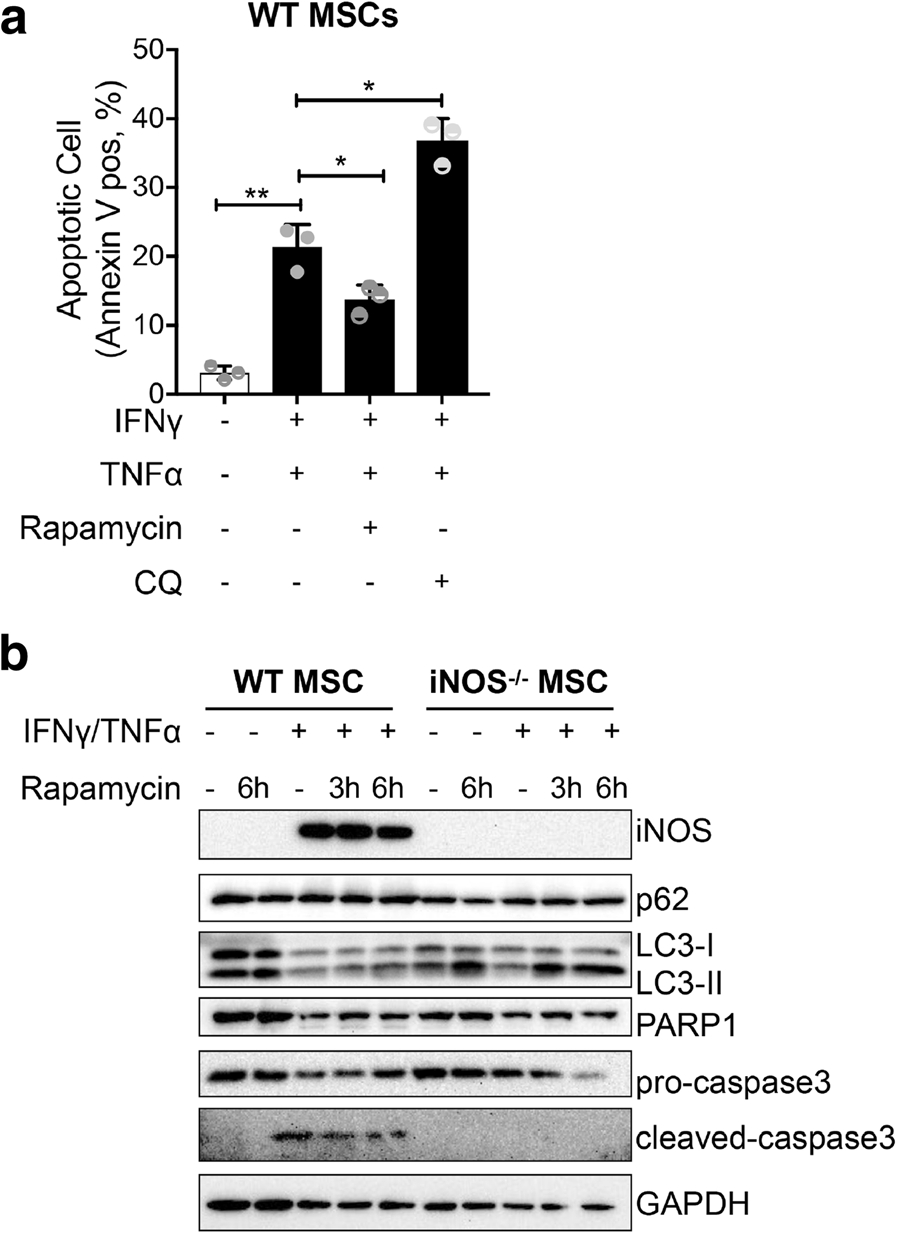
Blockade of autophagy by iNOS sensitizes BM-MSCs to apoptosis. **a** Apoptotic cells were analyzed using flow cytometry in MSCs treated or not (ctrl) for 48 h with IFNγ/TNFα (10 ng/ml each), alone or in combination with chloroquine (CQ), rapamycin. The percentage of Annexin V-positive cells relative to untreated controls is indicated in a bar chart. **b** Representative Western blot of LC3I/LC3II, p62, PARP, and Caspase-3 from wild-type BM-MSCs and iNOS^−/−^ BM-MSCs pretreated or not with IFNγ/TNFα (10 ng/ml each) for 16 h and treated with rapamycin for the indicated time

Autophagy is a catabolic process aimed at restoring energy homeostasis through self-digestion of intracellular proteins and organelles to survive under starvation. It also functions to resist other types of stress that cause damage in organelles, such as the endoplasmic reticulum (ER) or mitochondria [22]. Notably, NO has been reported to impair autophagy by inhibiting the activity of *S*-nitrosylation substrates, JNK1 and IKKβ [11]. We thus evaluated the impact of autophagy inhibition on the survival of MSCs with or without exposure to TNFα/IFNγ. Inhibition of autophagy through 16-h exposure to chloroquine (CQ: 20 μM) decreased MSC cell viability and enhanced MSC apoptosis (Fig. 5a). Stimulating autophagy using the mTORC1 inhibitor rapamycin (Rap: 100 nM), on the other hand, could protect BM-MSCs against IFNγ/TNFα-induced BM-MSC apoptosis (Fig. 5a). The processing of LC3-I into LC3-II marks autophagosome formation. LC3-II accumulation can be detected in two situations: (i) autophagic activity is stimulated and (ii) autophagic flux is blocked and vesicles accumulate [23]. Time-course of autophagy blockade using CQ allows monitoring of the autophagy flux. As expected, in control condition, autophagic flux blockade by CQ led to the accumulation of LC3-II and p62 due to the buildup of newly formed autophagosomes (Additional file 1: Figure S1). Further experiments were performed using rapamycin to stimulate autophagy. In absence of IFNγ/TNFα cytokines, rapamycin elevated LC3-II levels in iNOS^−/−^, but not in wild-type, BM-MSCs (Fig. 5b). The 12 h pre-treatment with IFNγ/TNFα cytokines abolished the effect of rapamycin on LC3-II in wild-type, but not in iNOS^−/−^, BM-MSCs (Fig. 5b), suggesting that the impairment of autophagic flux by cytokine exposure is dependent on the activity of iNOS. Augmentation of autophagy by rapamycin protected BM-MSCs against IFNγ/TNFα-induced BM-MSC apoptosis, as reflected by substantially attenuated activation of caspase-3 (Fig. 5b). These results suggest that NO may promote apoptosis of MSCs exposed to IFNγ/TNFα by impairing the pro-survival autophagy.

ER stress is also considered a major cause of apoptosis in many biological processes. We speculated that cytokines might induce ER overload and ER stress, resulting in the unfolded protein response (UPR)-triggered apoptosis of MSCs. As expected, treatment of MSCs with a combination of IFNγ and TNFα resulted in the upregulation of the levels of Atf4, Bip/GRP78, Chop, ERdj4/Dnajb9, and Xbp1, which are considered general ER stress biomarkers (Additional file 2: Figure S2a). Notably, ER stress induced by cytokines could be alleviated by NOS inhibitor, L-NMMA, as conveyed by downregulation of several general ER stress biomarkers (Additional file 2: Figure S2a). Similar results were also observed in Fas^−/−^ BM-MSCs (Additional file 2: Figure S2b). Autophagy may alleviate ER stress triggered by damaged organelles. To assess the relationship between ER stress and autophagy, we studied the expression of the known ER stress marker. IFNγ/TNFα-induced ER stress marker expressions were increased in presence of CQ, suggesting that blocking autophagy induced ER stress (Additional file 3: Figure S3). Interestingly, rapamycin exposure decreased IFNγ/TNFα-induced ER stress gene expression, indicating that increasing autophagic activity reduced ER stress (Additional file 3: Figure S3).

## Discussion

MSCs exhibit potent immunosuppressive and anti-inflammatory activities and have been demonstrated to have therapeutic effects in various diseases and in animal models of GvHD and autoimmune diseases including SLE, experimental autoimmune encephalomyelitis (EAE), and collagen-induced arthritis (CIA) [1, 3]. Additionally, MSCs are also being used in tissue and organ transplantation in several clinical trials, including islet, liver, and renal transplantation [4, 24, 25]. However, the results have often been inconsistent. Many issues remain unresolved. Among them is the varied response to MSC infusions in different patients affected by the same disease. It appears that MSCs can be therapeutically efficacious without being engrafted in the hosts. In fact, the vast majority of infused MSCs reside only transiently in the lungs before becoming undetectable within a few hours [6]. How those MSCs disappear in vivo remains largely elusive. When exposed to IFNγ/TNFα, mouse MSCs could undergo apoptosis mediated by Fas and were thus compromised in their bone-repairing function [7]. IFNγ/TNFα were also shown to induce autophagy in MSCs and inhibition of autophagy could augment the immunosuppressive effect on T cells by MSCs [8, 9]. However, survival of MSCs was recently shown to be dispensable for the immunosuppressive effect [10]. These studies suggest that a better understanding of the fate of MSC infusions and the mechanisms underlying MSC therapeutic activity would be highly desirable.

**Fig. 6:**
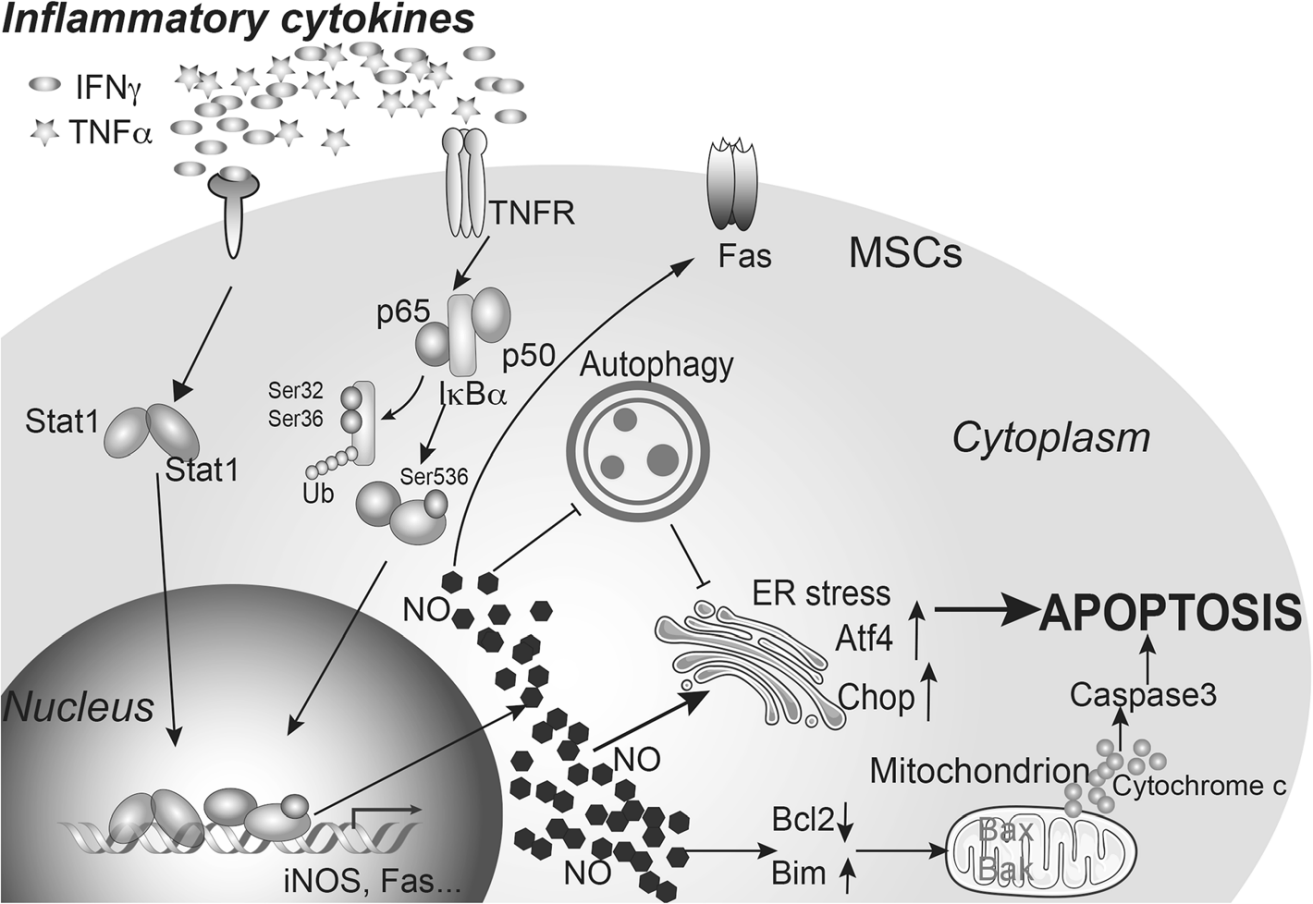
The role of iNOS in IFNγ/TNFα-induced apoptosis of MSCs. MSCs undergo apoptosis due to the synergistic action of IFNγ and TNFα under an inflammatory microenvironment. The pro-inflammatory cytokines IFNγ and TNFα induce apoptosis of mouse BM-MSCs in an NO-dependent manner. NO impairs autophagy in BM-MSCs and contributes to ER stress. NO also upregulates Fas and enhances Fas-mediated apoptosis

We here demonstrated that the apoptosis of mouse BM-MSCs triggered by the synergistic action of IFNγ and TNFα is mediated by NO. Importantly, we observed that IFNγ/TNFα also induced apoptosis in Fas^−/−^ MSCs in an NO-dependent manner, and Fas contributed to IFNγ/TNFα-induced cell death only when a Fas agonist was added (schematic representation described in Fig. 6). Moreover, IFNγ/TNFα-induced expression of Fas also depended on NO, and consequently, Fas agonist-induced cell death was greatly attenuated in iNOS^−/−^ MSCs. Our finding is thus in contrast to the previous report showing Fas pathway as a mediator of the cell death caused by the two cytokines [7]. Future studies are needed to resolve this discrepancy.

NO is a potent and pleiotropic free radical molecule that has been involved in a wide variety of physiological and pathophysiological functions [26]. NO and NO-derived reactive nitrogen species can interact with many receptors, ion channels, and enzymes [27]. NO is generated in low levels by two constitutive NOS (eNOS and nNOS) and in much greater levels by the iNOS [28]. NO is a ubiquitous cellular messenger molecule in the cardiovascular, nervous, and immune systems, where NO is capable of eliciting a multitude of physiological responses, such as blood flow regulation and tissue responses to hypoxia [29]. At high concentrations, NO inhibits TCR-induced T cell proliferation and cytokine production [30]. However, there has been a long debate about the specific role that NO plays in apoptosis. It has been shown that NO could participate in the apoptosis process by either inhibiting or promoting some apoptotic events. Several studies have referred that NO is a novel and potent inhibitor of apoptosis. Endogenous NO synthesis or exposure to low levels of NO donors has been demonstrated to inhibit apoptosis in human B lymphocytes, splenocytes, eosinophils, and endothelial cells [31–34]. NOS inhibitors have also been directed toward the specific disruption of the Fas-induced apoptotic mechanism. Basal NOS activity in human leukocytes has been revealed to inhibit Fas-induced apoptosis via a cGMP-independent mechanism and further inhibition of caspase activation [35, 36]. Conversely, an excess of NO is believed to play a causal role in demise of several type of cells, including neuronal cells [13], T cells [14], dendritic cells [15], and microglial cells [16]. The involvement of Fas/FasL system and signal transduction pathway in NO-induced apoptosis in human lymphoid cells has been examined, suggesting that NO triggers the death receptor system by regulating the expression of ligands involved in apoptosis [37]. Herein, our results with mouse MSCs clearly demonstrate that NO, far from protecting MSCs from Fas-induced apoptosis, synergized with the Fas agonist antibody Jo2 in the induction of apoptosis. The dichotomy in the function of NO may reflect divergence among different cell types and needs to be considered when making strategies toward the use of NO donors as therapeutic agents.

Autophagy, the basic catabolic process, occurs at basal levels in most tissues and contributes to routine cell recycling by lysosomes. During this process, substances in the cytoplasm are phagocytosed by autophagosomes, which are transported to the lysosomes for degradation. The degradation products can be reused in the syntheses of macromolecules and in energetic metabolism. Autophagy plays an important role in cell survival process [38]. Early studies suggested that autophagy serves as a cell survival mechanism in some pathological processes via its suppressive role in necroptosis and PARP-mediated apoptosis during unfavorable growth conditions or cellular stress [39]. Autophagy plays an important role in suppressing apoptosis in retinal ganglion cells. It has also been observed that activation of autophagy could promote retinal ganglion cell survival and that inhibition of autophagy can reduce cell survival during optic nerve degeneration [40]. ER plays a major role in the synthesis, folding, and structural maturation of more than a third of all proteins made in the cell [41]. ER stress is caused by the accumulation of misfolded proteins in the ER, which triggers an adaptive program. Autophagy may alleviate the ER stress triggered by damaged organelles [42]. In this study, we also showed that the generation of NO in MSCs stimulated by IFNγ/TNFα impairs autophagy, which aggravates ER stress and promotes apoptosis. The finding that NO can impair pro-survival autophagy also renders support to this notion [11]. Collectively, our data indicate that inhibition of autophagy by NO induced by inflammatory cytokines plays a pro-apoptotic role in MSCs and it might not benefit the survival of MSCs when they are transplanted for inflammatory diseases.

Our study only focuses on the effects of NO induced by inflammatory cytokines on the fate of MSCs in vitro, whether NO-induced apoptosis of mouse MSCs accounts for the disappearance of MSCs in vivo after infusions in inflammatory diseases remains unclear. However, while deletion or inhibition of iNOS can potentially prolong the survival of mouse MSCs in vivo, the immunosuppressive function of MSCs thus obtained may also be compromised because NO is essential for the inhibition of the T cells.

## Conclusions

Our studies show that the pro-inflammatory cytokines IFNγ and TNFα synergistically induce apoptosis of mouse BM-MSCs in an NO-dependent manner. NO was found to impair autophagy in BM-MSCs and to contribute to ER stress. NO also upregulates Fas and enhances Fas-mediated apoptosis. These findings provide an important insight into the mechanism by which mouse MSCs undergo apoptosis in the presence of pro-inflammatory cytokines and should bear implications in MSC-based therapy.

## Additional files

Additional file 1: **Figure S1.** Effect of a combination of IFNγ and TNFα on the inhibition of autophagy in MSCs. Representative Western blot of LC3I/LC3II and p62 from both wild-type and iNOS^−/−^ BM-MSCs pretreated or not with IFNγ/TNFα (10 ng/ml each) for 16 h and treated with chloroquine (CQ) for the indicated time. (TIF 212 kb)

Additional file 2: **Figure S2.** IFNγ/TNFα induce ER stress in BM-MSCs dependently iNOS activity. (a and b) Both wide type and Fas^−/−^, BM-MSCs were treated with IFNγ/TNFα (10 ng/ml each) in the absence or presence (1 h pre-incubation) of L-NMMA (1 mM) for 24 h, ER stress-related transcripts were quantified by real-time PCR. (TIF 316 kb)

Additional file 3: **Figure S3.** ER stress was upregulated by blocking autophagy. MSCs were treated or not (mock) for 48 h with IFNγ/TNFα (10 ng/ml each), alone or in combination with chloroquine, rapamycin. ER stress-related gene transcripts were quantified by real-time PCR. (TIF 645 kb)

## Abbreviations

AIF: Apoptosis-inducing factor
CIA: Collagen-induced arthritis
CQ: Chloroquine
EAE: Experimental autoimmune encephalomyelitis
ER: Endoplasmic reticulum
GvHD: Graft-versus-host disease
iNOS: Inducible nitric oxide synthase
L-NMMA: N^G^-monomethyl-L-arginine
MSCs: Mesenchymal stem/stromal cells
NO: Nitric oxide
SLE: Systemic lupus erythematosus
SNAP: *S*-Nitroso-*N*-acetyl-penicillamine
UPR: Unfolded protein response
YY1: Yin Yang 1
