# Supplementary figures and images for: IFNγ and TNFα synergistically induce apoptosis of mesenchymal stem/stromal cells via the induction of nitric oxide
Source: Stem Cell Res Ther. 2019 Jan 11;10:18. doi: 10.1186/s13287-018-1102-z (PMC6330503; doi:10.1186/s13287-018-1102-z)

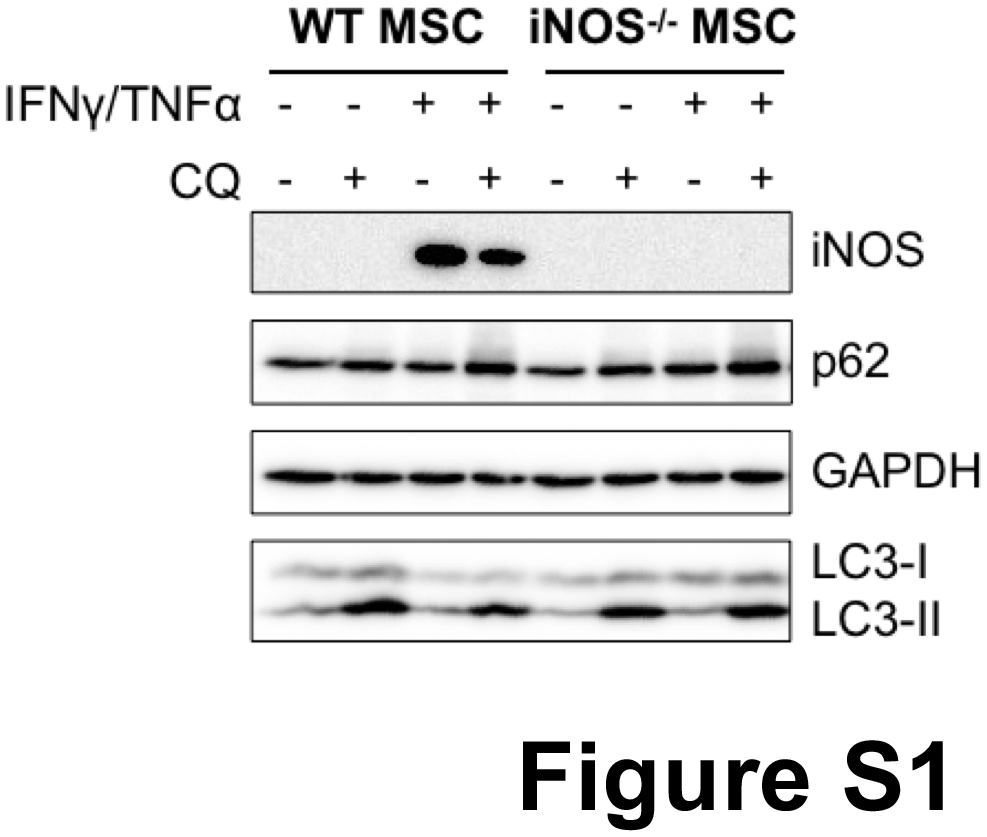

Supplement: Supplementary file 1 — Figure S1. Effect of a combination of IFNγ and TNFα on the inhibition of autophagy in MSCs. Representative Western blot of LC3I/LC3II and p62 from both wild-type and iNOS−/− BM-MSCs pretreated or not with IFNγ/TNFα (10 ng/ml each) for 16 h and treated with chloroquine (CQ) for the indicated time. (TIF 212 kb) [file 13287_2018_1102_MOESM1_ESM.tif]

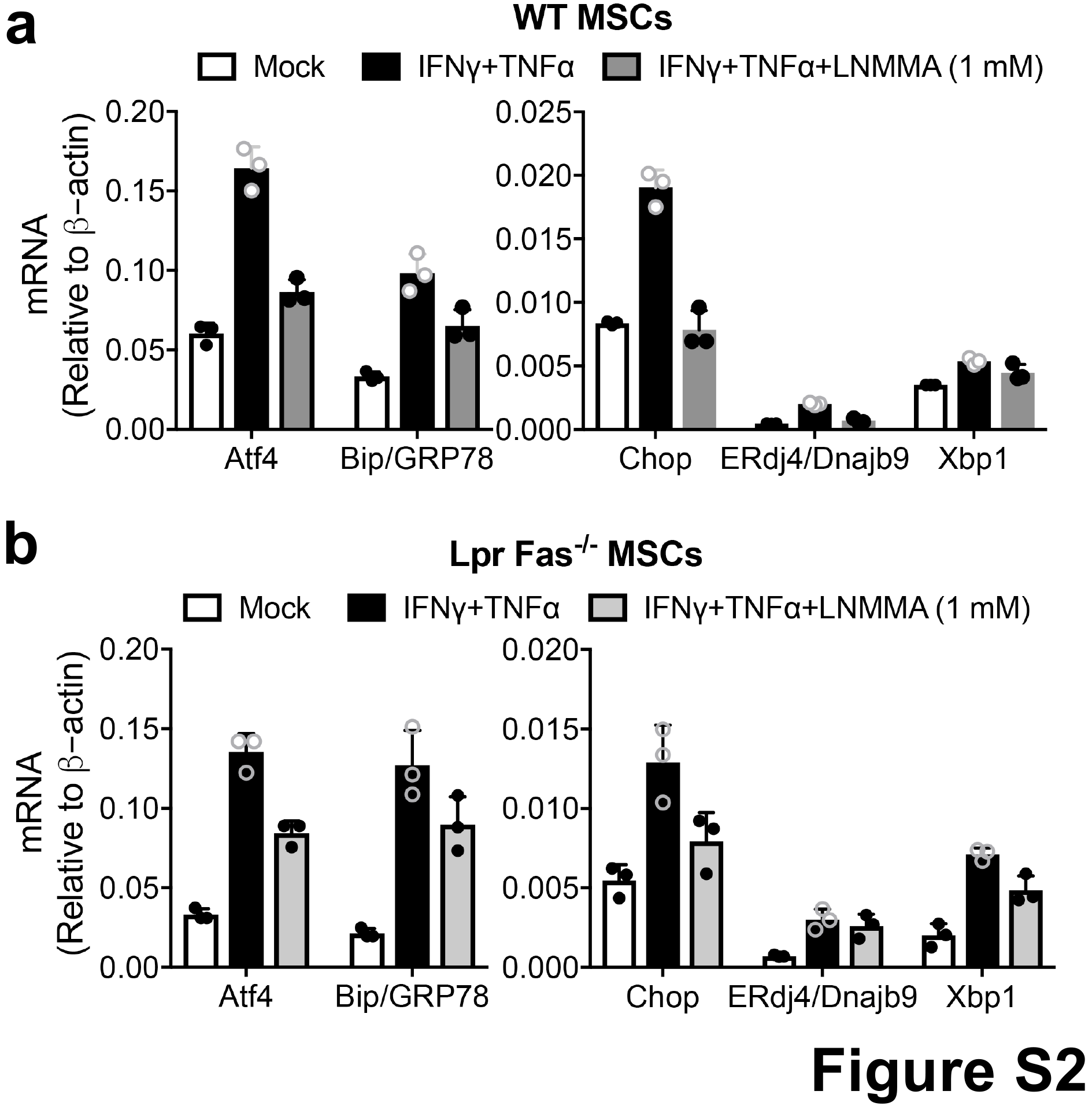

Supplement: Supplementary file 2 — Figure S2. IFNγ/TNFα induce ER stress in BM-MSCs dependently iNOS activity. (a and b) Both wide type and Fas−/−, BM-MSCs were treated with IFNγ/TNFα (10 ng/ml each) in the absence or presence (1 h pre-incubation) of L-NMMA (1 mM) for 24 h, ER stress-related transcripts were quantified by real-time PCR. (TIF 316 kb) [file 13287_2018_1102_MOESM2_ESM.tif]

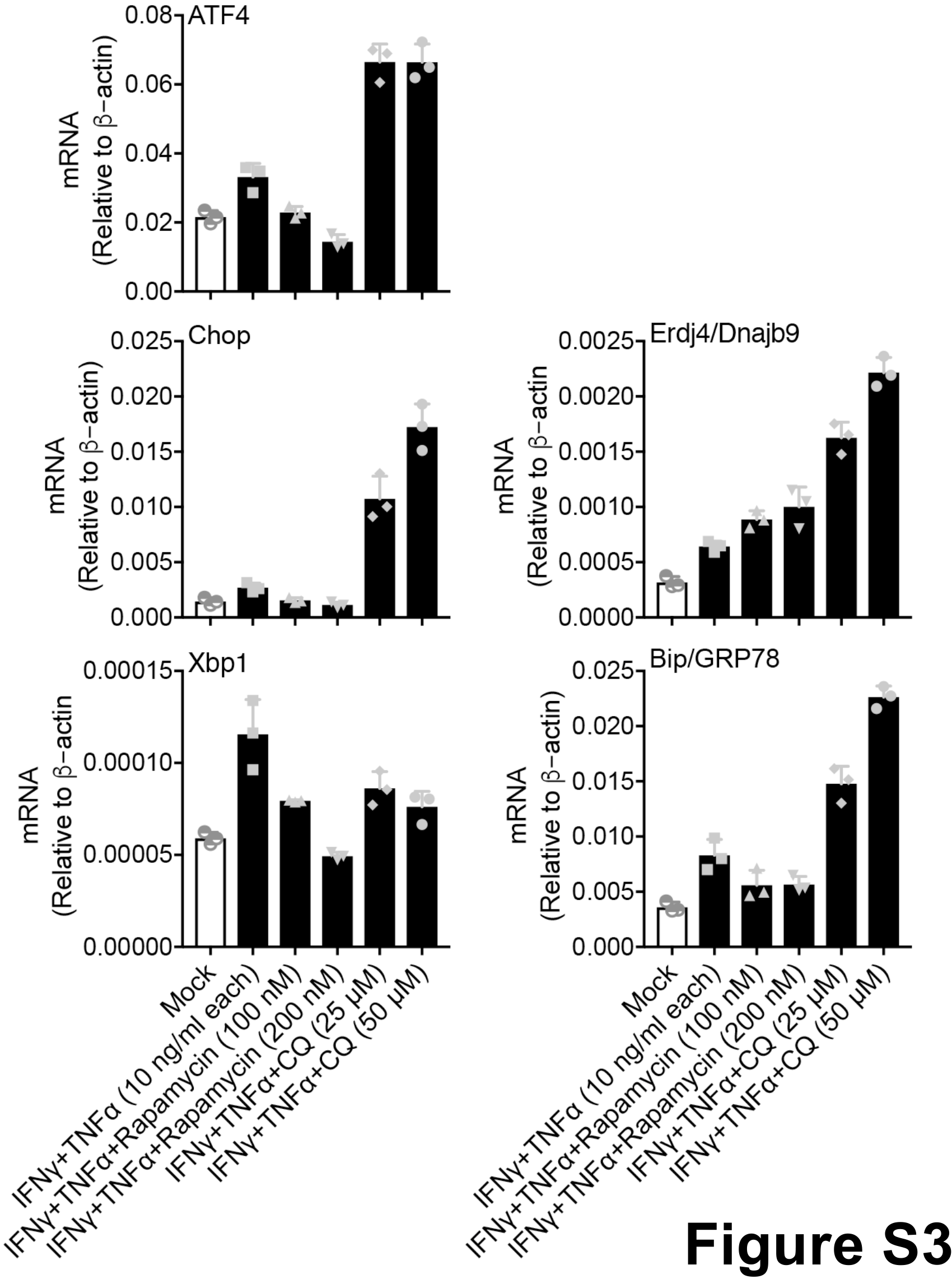

Supplement: Supplementary file 3 — Figure S3. ER stress was upregulated by blocking autophagy. MSCs were treated or not (mock) for 48 h with IFNγ/TNFα (10 ng/ml each), alone or in combination with chloroquine, rapamycin. ER stress-related gene transcripts were quantified by real-time PCR. (TIF 645 kb) [file 13287_2018_1102_MOESM3_ESM.tif]
